# Mitochondrial inhibitors: a new horizon in breast cancer therapy

**DOI:** 10.3389/fphar.2024.1421905

**Published:** 2024-07-04

**Authors:** Yalan Yan, Sijie Li, Lanqian Su, Xinrui Tang, Xiaoyan Chen, Xiang Gu, Guanhu Yang, Hao Chi, Shangke Huang

**Affiliations:** ^1^ Clinical Medical College, Southwest Medical University, Luzhou, China; ^2^ Department of Oncology, The Affiliated Hospital of Southwest Medical University, Luzhou, China; ^3^ Paediatrics Department, Southwest Medical University, Luzhou, China; ^4^ The First Affiliated Hospital of Wenzhou Medical University, Wenzhou, China; ^5^ Biology Department, Southern Methodist University, Dallas, TX, United States; ^6^ Department of Specialty Medicine, Ohio University, Athens, OH, United States

**Keywords:** mitochondrial inhibitors, breast cancer, cancer subtype, tumor progression, metabolic reprogramming

## Abstract

Breast cancer, due to resistance to standard therapies such as endocrine therapy, anti-HER2 therapy and chemotherapy, continues to pose a major health challenge. A growing body of research emphasizes the heterogeneity and plasticity of metabolism in breast cancer. Because differences in subtypes exhibit a bias toward metabolic pathways, targeting mitochondrial inhibitors shows great potential as stand-alone or adjuvant cancer therapies. Multiple therapeutic candidates are currently in various stages of preclinical studies and clinical openings. However, specific inhibitors have been shown to face multiple challenges (e.g., single metabolic therapies, mitochondrial structure and enzymes, etc.), and combining with standard therapies or targeting multiple metabolic pathways may be necessary. In this paper, we review the critical role of mitochondrial metabolic functions, including oxidative phosphorylation (OXPHOS), the tricarboxylic acid cycle, and fatty acid and amino acid metabolism, in metabolic reprogramming of breast cancer cells. In addition, we outline the impact of mitochondrial dysfunction on metabolic pathways in different subtypes of breast cancer and mitochondrial inhibitors targeting different metabolic pathways, aiming to provide additional ideas for the development of mitochondrial inhibitors and to improve the efficacy of existing therapies for breast cancer.

## 1 Introduction

Breast cancer (BC) is one of the most common malignancies worldwide, and triple-negative breast cancer (TNBC) is considered to be one of the most aggressive subtypes (Cocco et al., 2020; Obidiro et al., 2023). According to global data for 2020, there are nearly 2.26 million new cases of breast cancer and approximately 680,000 deaths (Cocco et al., 2020). However, the standard of care (SOC) for BC, including endocrine therapy for estrogen receptor-alpha (ERα) positive, anti-HER2 monoclonal antibody therapy for human epidermal growth factor receptor-2 (HER2) positive, and chemotherapy for TNBC subtypes, often face limitations in clinical practice due to drug tolerance (Waks and Winer, 2019; Barzaman et al., 2020). Studies have shown that altered metabolism is a major contributor to drug resistance. In addition, recent studies have indicated that mitochondrial inhibitors have great potential for use in cancer treatment, either alone or in combination with other cancer therapies (Pollak, 2012; Wheaton et al., 2014). All of these advances provide new perspectives and directions for the treatment of TNBC as well as drug-resistant breast cancer.

Mitochondria are known as the “energy factories” of the cell, capable of incorporating OXPHOS, TCA cycle, fatty acid metabolism and amino acid metabolism (Singh et al., 2017; Monzel et al., 2023). Enzymatic dysfunctions and disruptions in the electron transport chain (ECT) may lead to alterations of metabolism and redox balance, which in turn may lead to abnormal cell death and alterations in the redox balance alterations, which in turn trigger aberrant cell death and the development of resistance to tumor therapy (DiMauro and Schon, 2003; Chaiyarit and Thongboonkerd, 2020; Missiroli et al., 2020; Zhu et al., 2023). In this context, a large number of studies have revealed a key feature of tumor metabolism - even in the presence of sufficient oxygen and normal mitochondrial function, tumor cells exhibit a dependence on enhanced glucose uptake and aerobic glycolysis. Tumor cells exhibit a dependence on glucose uptake and aerobic glycolysis even in the presence of sufficient oxygen and normal mitochondrial function, a phenomenon known as the‘Warburg effect’, reflecting an aberrant metabolic adaptation of tumor cells (Koppenol et al., 2011; Lu, 2019).

Therefore, targeting mitochondrial inhibitors, either alone or in combination with standard therapy, is a rational and attractive strategy (Lin et al., 2024). Currently, a variety of inhibitors targeting mitochondrial oxidative phosphorylation are in development, including classical inhibitors such as Metformin and ME-344 (Quintela-Fandino et al., 2020; Sahu et al., 2022), and newly discovered compounds such as IACS010759 (Tsuji et al., 2020; Lu et al., 2021), which are undergoing translational clinical studies.

## 2 Role of mitochondria in breast cancer

**FIGURE 1:**
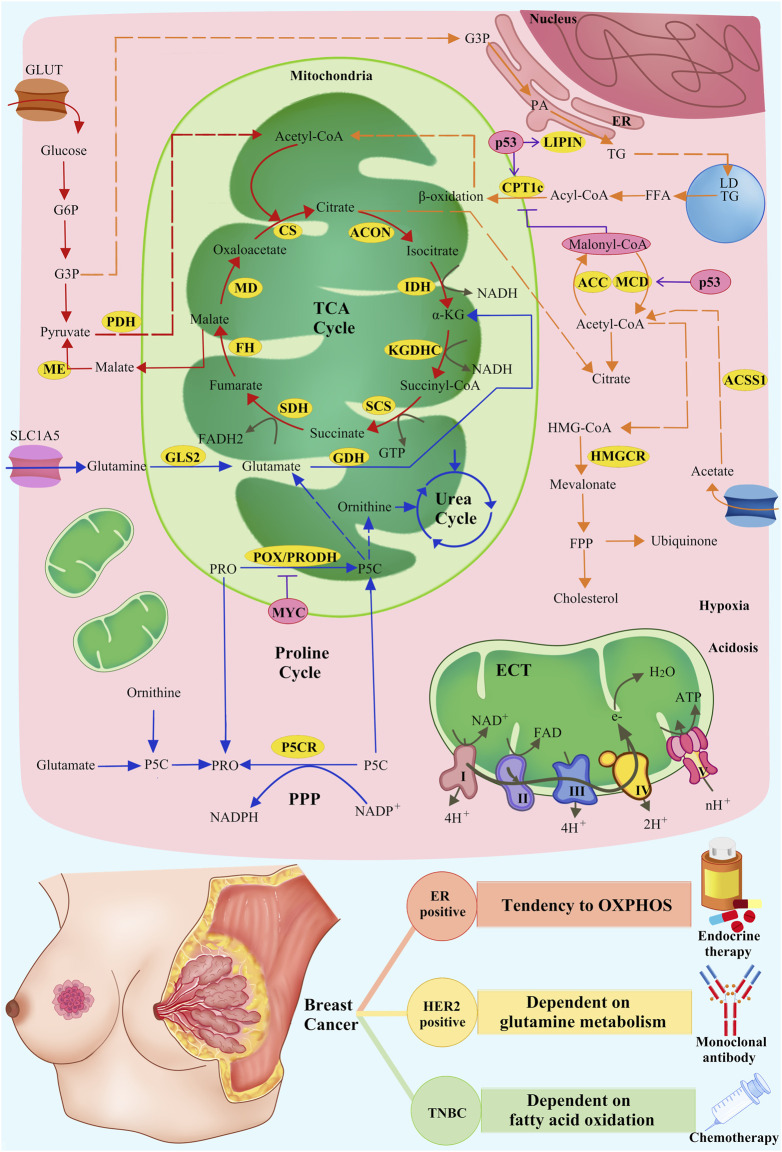
Metabolic mechanisms of mitochondrial inhibitors and SOC of breast cancer. Mitochondria-related pathways of glucose, lipid, and amino acid metabolism and OXPHOS (red arrows: glucose metabolism; orange arrows: lipid metabolism; blue arrows: amino acid metabolism; grey arrows: OXPHOS), related enzymes in metabolism (yellow cycles: metabolism-related enzymes), and the regulation of related enzymes by intrinsic and extrinsic factors (pink cycles: intrinsic and extrinsic factors; purple T symbols: inhibiting enzymes; purple arrows: promoting enzymes) are shown.

Mitochondria are key organelles involved in energy production and cellular metabolism, especially in cancer cell metabolism, such as glucose metabolism, lipid metabolism and amino acid metabolism (Liu et al., 2023; Liu et al., 2024) (Figure 1). In BC, these normal metabolic functions within the mitochondria are significantly altered due to internal and external factors, resulting in the so-called “metabolic reprogramming” (Wang et al., 2020). This metabolic reprogramming is a new metabolic strategy adopted by tumor cells in response to changing microenvironmental and various stress conditions, aiming at supporting their proliferation, survival and invasiveness, thereby promoting tumor progression.

Intrinsic factors involve transcription factors such as MYC, ER, p53, BCAH, and HIF-1a, whereas extrinsic factors include reactive oxygen stress and high-fat diets, among others (Lewoniewska et al., 2021; Mao and Jiang, 2023). These factors exert selective pressures on tumor cells, leading to the fact that only those cells that have been transformed through adaptive metabolism can survive and reproduce (Cruz-Leite et al., 2023). In a study by Qianlu Yang et al., the tumour heterogeneity of type A and type B breast cancers was revealed by using metabolomics and transcriptomics techniques. This finding further confirms that different subtypes of breast cancer have different metabolic properties, which has important implications for therapeutic strategies (Mao and Jin, 2019; Yang et al., 2024) (Table 1).

**TABLE 1:** Mitochondrial inhibitors: Targeting metabolism in disease management.

Metabolism	Intervention	Target	Disease type	References
Lipid metabolism	Etomoxir	CPT1	Breast cancer	Hou et al. (2020)
	Trimetazidine	Beta-oxidation	Heart failure	van de Bovenkamp et al. (2023)
Amino acid metabolism	CB839	GLS1	Breast cancer, multiple myeloma, rectal cancer	Demas et al. (2019), Zhao et al. (2020), Hong et al. (2022)
	Canagliflozin	GDH	breast cancer	Papadopoli et al. (2021)
Glucose metabolism	Enasidenib	IDH	AML	Chen et al. (2023)
	HH-2301	IDH1	Advanced cholangiocarcinoma, chondrosarcoma and glioma	Wang et al. (2022)
	Dichloroacetate	PDK	NSCLC, metastatic breast cancer	Zhang et al. (2018)
	CPI-613	PDH	Pancreatic cancer, biliary tract cancer	Sun (1981), Mohan et al. (2023)

	ME-344	Complex I	Breast cancer	Quintela-Fandino et al. (2020)
	Metformin	Complex I	Breast cancer, thyroid cancer	Thakur et al. (2018), Sahu et al. (2022)
	FRV-1	Complex I	Breast cancer	Monroy-Cárdenas et al. (2023)
	IACS-010759	Complex I	AML	Tsuji et al. (2020), Lu et al. (2021)
	DX3-213B	Complex I	Pancreatic cancer	Xue et al. (2022)
	Mito-LND	Complex II	Lung cancer, glioblastoma	Cheng et al. (2019), Guo et al. (2023)
	Oligomycin A	Complex V (ATP synthase)	Breast cancer	Gale et al. (2020)

## 3 Targeting lipid metabolism

Lipid metabolism consists of a complex series of molecular processes involving lipid uptake, *denovo* synthesis, and catabolism, with fatty acid oxidation (FAO) in the mitochondria being particularly critical (Fu et al., 2020). Within the mitochondria, fatty acids are converted to acetyl coenzyme A (Ac-CoA) via FAO, and Ac-CoA subsequently enters the TCA cycle and the oxidative phosphorylation process to produce ATP and key oxidative reducing coenzymes, such as NADH and FADH_2_, which provide the necessary energy to maintain normal cellular function (Badr et al., 2020). In breast cancer cells, then, specific reprogramming of lipid metabolism occurs to meet the demands of their rapid growth.

Internal factors Oncogenic factor p53 regulates metabolism through its non-classical pathway and inhibits tumorigenesis. Wild-type p53 (wt p53) promotes FAO and inhibits tumor proliferation by up-regulating the expression of carnitine palmitoyltransferase 1C (CPT1C), malonyl coenzyme A decarboxylase (MCD), and lipoprotein 1 (LPIN1) (Zhuang et al., 2019; Fadó et al., 2023; Mao and Jiang, 2023; Wang et al., 2023; Zheng et al., 2023). Mutant p53 reduces phosphorylation of acetyl coenzyme A carboxylase (ACC) by inhibiting AMP-activated protein kinase (AMPK), leading to increased malonyl coenzyme A levels and CPT1 receives inhibition, resulting in decreased FAO levels as well as enhancement of the lipid synthesis pathway (Zhou et al., 2014; Herzig and Shaw, 2018).

In addition, p53 interacts with intrinsic factors, such as SREBP, and extrinsic factors, such as hypoxia and high-fat environments, to regulate mitochondrial lipid metabolism. In breast cancer, mutant p53 increases cholesterol synthesis and ubiquinone production by activating SREBP2 to promote the mevalonate pathway (Dai et al., 2022; Laka et al., 2022). Ubiquinone is not only involved in electron transfer within mitochondria, but also acts as an antioxidant to adapt tumor cells to changes in external factors such as increased reactive oxygen species (ROS), and these functions promote tumorigenesis metastasis (Laka et al., 2022). In hypoxia or nutritional deficiencies, cancer cells can promote the conversion of cholesterol into cholesterol via acetyl coenzyme A synthetase (ACSS) one and 2 promote the transformation of acetate to Ac-CoA, which maintains TCA cycling and energy production and supports cancer cell survival (Schug et al., 2015; Gao et al., 2016). In patients with TNBC on a high-fat diet, tumor cells promote ATP production by enhancing mitochondrial FAO (Dai et al., 2022).

Different subtypes of breast cancer show significant differences in lipid metabolism (Dai et al., 2022). For example, in HER2^+^ BC, higher expression levels of CPT1A and fatty acid synthase (FASN) show a strong dependence on lipid metabolism. Research has demonstrated that FAO constitutes a significant metabolic pathway in TNBC, and is correlated with the activation of Src signaling (Ahn et al., 2024). Moreover, it has been found that fatty acid oxidation is required for metastasis in TNBC (Madan et al., 2021). In addition, metabolic reprogramming leads to an increase in FAO in ER^+^ breast cancers, which improves resistance to resistance to endocrine therapy (Yan et al., 2023).

Therefore, personalized therapeutic strategies by knocking down the CPT1 gene or using FAO inhibitors for specific subtypes can effectively enhance therapeutic efficacy and increase sensitivity of tumor cells to treatment (Li et al., 2021). Malonyl CoA has an inhibitory effect on CPT1, which, in turn, inhibits FAO (Zhelev et al., 2022). Based on this, it is not surprising that the use of malonyl coenzyme A decarboxylase MCD inhibitors to increase malonyl CoA content has emerged as a potential metabolic pathway strategy for exploring heart failure therapies (Wang et al., 2019). In addition, Etomoxir, the most commonly used CPT1 inhibitor, had a significant inhibitory effect on MYC-induced mammary carcinoma mice (Hou et al., 2020).

## 4 Targeting amino acid metabolism

Tumor cells also adapt to internal and external selective pressures and material-energy demands by reprogramming amino acid metabolism to accommodate these selective pressures and demands. Among them, proline metabolism and glutamate metabolism play important roles in metabolic reprogramming in cancer (Liu et al., 2012; Zhou et al., 2019; Guo and Wu, 2020).

Proline oxidase/proline dehydrogenase (POX/PRODH), an enzyme associated with the inner mitochondrial membrane, plays an essential role in breast cancer cell growth and metastasis by converting proline to pyrroline-5-carboxylate (P5C) and generating FADH_2_, which provides electrons for the ECT and promotes ATP production (Zhou et al., 2019). In BC, the intrinsic factor p53 directly regulates PRODH/POX transcription, whereas MYC indirectly affects PRODH/POX by stimulating the expression of miR-23b (Lewoniewska et al., 2021). Enhanced expression of PRODH has been observed in metastatic tumors relative to primary tumors among patients with BC (Tanner et al., 2018). In orthotopic 4T1 mouse model, lung metastasis of tumors was reduced by targeting PRODH and did not damage normal tissue cells (Tanner et al., 2018).

Glutamate and ornithine are the main sources of proline, which can also be synthesized from proline in mitochondria, thus linking proline metabolism to the TCA cycle and the urea cycle (Burke et al., 2020). The p53 promotes the conversion of glutamine to glutamate by upregulating glutaminase 2 (GLS2). Glutamate is the precursor of α-ketoglutarate (α-KG), a key component of the TCA cycle, thereby fuelling the TCA cycle and maintaining cellular redox homeostasis (Lukey et al., 2019). In drug-resistant breast cancer MYC is overexpressed, glutamine transporter proteins SLC1A5 and GLS are upregulated, and glutamate metabolism is markedly enhanced to promote proliferation of drug-resistant tumor cells. Inhibition of MYC, SLC1A5 and GLS effectively attenuated the proliferation of drug-resistant cell (Cunha et al., 2023).

Different degrees of amino acid reprogramming were demonstrated in different types of BC. Specifically, the HER2^+^ subtype is one of the most glutamine metabolism-dependent subtypes, and the elevated levels of glutamine transporter protein (SLC1A5) and GLS transcripts in HER2^+^ breast cancers increased their dependence on glutamine metabolism, thus identifying new therapeutic targets for HER2^+^ breast cancers (Lv et al., 2022). In addition, the overexpression of GLS in TNBC, which is highly resistant to Glutamine catabolism-targeted therapy was sensitive, with specific metabolic levels expressed as a low glutamine/glutamate ratio (Wang et al., 2020). Whereas in ER^+^ BC, compared to other subtypes of breast cancer, a low glutamate level was expressed (Cha et al., 2018).

In treatment, according to the metabolic profiles of different subtypes, inhibitors targeting amino acid metabolism can be used for precision therapy to modulate tumor cell proliferation, metastasis, and drug resistance (Hong et al., 2022). Studies have shown that targeting glutamine metabolism enhances the sensitivity of TNBC to platinum-based chemotherapy. In addition, the glutaminase inhibitor CB839 effectively inhibited tumor growth of TNBC cells (Demas et al., 2019).

## 5 Targeting glucose metabolism

Glucose is the most common source of energy for mammalian cells and it can be converted to pyruvate by glycolysis. Under hypoxic conditions, pyruvate undergoes reduction to lactate mediated by lactate dehydrogenase (LDH). Conversely, in an oxygen-rich environment, pyruvate is transported into the mitochondria where it is decarboxylated by the pyruvate dehydrogenase (PDH) complex, leading to the production of Ac-CoA. The oxidation of Ac-CoA occurs via the TCA cycle, involving a series of key enzymes, and ultimately produces CO_2_, H_2_O, and bioenergetic products GTP, NADH, and FADH_2_ (Krebs, 1970; Freire et al., 2014). In summary, the TCA cycle represents the final polymerization pathway for the oxidation of lipids, carbohydrates, and amino acids (Akram, 2014).

Within tumor mitochondria, the TCA cycle often shows abnormalities in key enzymes of metabolism, which in turn contributes to the reprogramming of sugar metabolism in tumor cells (Nie et al., 2020). In addition, abnormalities in the TCA cycle further lead to abnormalities in mitochondrial oxidative phosphorylation of ATP-producing mitochondria (mtOXPHOS), and finally, have an impact on the proliferation, growth and metastasis of tumor cells (Ghilardi et al., 2022; Passaniti et al., 2022).

In tumor cells, glucose is biased toward the glycolytic pathway due to the phosphorylation of PDH by pyruvate dehydrogenase kinases (PDKs). Inhibition of PDKs activity not only blocks this metabolic pathway, but also activates mitochondrial oxidative metabolism and induces apoptosis. Studies have shown that breast cancer cells enhance survival by enhancing glycolysis, but activation of PDH restores glucose oxidation, increases tumor cell sensitivity to anaerobiosis, and reduces metastatic potential (Kamarajugadda et al., 2012).

Mutations of isocitrate dehydrogenase (IDH) one and 2 impair the decarboxylation of α-KG to isocitrate and enhance the production of 2-hydroxyglutarate (2HG) (Jane et al., 2023). This enzymatic disruption leads to elevated DNA methylation levels, a phenomenon frequently observed in diseases such as acute myeloid leukemia (AML) (Wilde et al., 2019; Pei et al., 2023).

Succinate dehydrogenase (SDH) and fumarate hydratase (FH) are key enzymes in the TCA cycle with concomitant oncogenic activity (Riemann et al., 2019; Muralidharan et al., 2022). In BC, SDH inhibits tumor cell growth and metastasis by suppressing EMT (Røsland et al., 2019). Meanwhile, FH has also been found to be absent or downregulated in its expression in breast cancer (Gonçalves et al., 2018; Riemann et al., 2019).

In addition, malic enzyme (ME2), which is involved in the replenishment response of the TCA cycle, correlates with the levels of HIF-1α in breast cancer and affects cell proliferation and metastasis (You et al., 2021). A liposomal nano-formulation delivering the Fenton’s catalyst, copper oleate, and the HIF-1 inhibitor, acridinium flavonoids (ACF), has been reported to be useful for breast cancer treatment, which further emphasizes the role of HIF-1α in the regulation of BC (Guo et al., 2021).

Abnormalities in these TCA cycle-related enzymes affect the entry of NADH, FADH_2_ into mtOXPHOS. The mtOXPHOS involves five complexes located on the inner mitochondrial membrane, which together make up the, ETC (Fernandez-Vizarra and Zeviani, 2021). During rapid proliferation, cancer cells exposed to hypoxic conditions often exhibit a metabolic transition from OXPHOS to glycolysis (Pal et al., 2022). A decrease in OXPHOS has been commonly described in BC cells. In MDA-MB-231 cells, TNF-α was found to reduce the activity of complex I (Shinde et al., 2021). In addition, in TNBC cells, mitochondrial respiratory capacity was significantly reduced due to downregulation of the expression of complexes I and V (Guha et al., 2018). However, it has been shown that inhibition of OXPHOS can target tumor stem cells, inhibit cellular dependence on OXPHOS, and reduce tumor cell survival and proliferation (Liu et al., 2023). Overall, targeting OXPHOS appears to be a promising approach to limiting metastasis in breast cancer.

For glucose metabolism, different cancers have different glycolytic-OXPHOS propensities. ER^+^ breast cancer has been shown to exhibit an intermediate metabolic phenotype in the glycolytic-OXPHOS spectrum. However, ER^+^ BC is more dependent on OXPHOS than TNBC (Xu et al., 2020). In HER2^+^ BC, mitochondrial HER2 tyrosine kinase activity is activated, which not only stimulates oxidative phosphorylation, but also accelerates glycolysis (Schlam and Swain, 2021). In terms of proline, metastatic breast cancer metabolizes proline more vigorously than primary breast cancer, and TNBC metastasizes fastest compared to other subtypes of breast cancer (Tanner et al., 2018; Jiang et al., 2024).

These findings emphasize the importance of reprogramming of glucose metabolism in cancer development and provide a theoretical basis for anticancer strategies with mitochondrial inhibitors. IDH1 and IDH2 mutations may also occur in cholangiocarcinomas, melanomas, prostate cancers, lung cancers, breast cancers, and colorectal cancers (McBrayer et al., 2018; Scotlandi et al., 2020). It has been found that IDH inhibitors. Enasidenib and ivosidenib have been approved for the treatment of BC (Chen et al., 2023). In addition, Dichloroacetate, a PDK inhibitor, contributes to the increase of PDH activity, and has been reported to have significant antimicrobial activity against NSCLC and metastatic breast cancer. ME-344 inhibits mitochondrial oxidative phosphorylation complex I and has been used as a single agent in a phase I trial for the treatment of patients with refractory solid tumors (Quintela-Fandino et al., 2020). In proline metabolism, rapamycin promotes mitochondrial autophagy in POX-dependent cancer cells by enhancing POX activity (Huynh et al., 2020), and HDAC inhibitors (TSA/SAHA) significantly increase POX expression and autophagy in TNBC cells by activating AMPK (McBrayer et al., 2018).

## 6 Discussion

With the deeper knowledge of one of the tumor hallmarks, namely, metabolic reprogramming, the field of targeting multiple metabolic pathways within the mitochondria has revived and many drugs have entered clinical practice or preclinical studies. Among them, mitochondrial inhibitors have a wide range of applications in the study and treatment of many diseases, especially in the study of mitochondrial disorders, metabolic syndromes, neurodegenerative disorders, and cancer. However, due to the necessity of mitochondria in normal cellular function, mitochondrial inhibitors can often have many adverse effects on normal cells. For example, IACS-010759, an inhibitor of complex I, showed side effects such as elevated lactate levels and neurotoxicity in a phase I clinical study (Yap et al., 2023). Therefore, monitoring the toxicity of antitumor drugs such as mitochondrial inhibitors to avoid damage to normal cells is a major concern.

In addition, the special structure of mitochondria itself is also a difficult point for the development of mitochondrial inhibitors. Compared with the permeability of the outer mitochondrial membrane, the inner mitochondrial membrane sets up a barrier for the flow of small molecule (Musicco et al., 2023). Therefore, the development of compounds targeting intracellular mitochondrial membrane transporter proteins may be an effective option.

In addition to this, key enzymes in mitochondrial metabolism often have multiple isoforms with high similarity, such as GLS one and 2, pyruvate kinase (PKM) 1 and 2, and hexokinase (HK) 1 and 2 (Sainero-Alcolado et al., 2022; Ma et al., 2023). Besides, mitochondrial enzymes exhibit plasticity in cancer progression. In a single-cell assessment of gene expression of tumor metabolic enzymes, mitochondrial enzymes were found to exhibit the highest variability in the same tumor (Xiao et al., 2019). Coupled with the fact that the intracellular and extracellular microenvironment can vary depending on tumor type or subtype, cancer cells exhibit metabolic heterogeneity in the face of changes in internal and external factors (Fendt et al., 2020). The development of mitochondrial inhibitor presents significant challenges. Strategies for metabolic monotherapy include targeting multiple metabolic pathways simultaneously or using drugs for specific metabolic pathways in combination with standard therapies. This approach provides a reasonable option for cancer treatment. Critically, effective metabolic therapies require the integration of a multi-omics approach and the use of advanced technologies including metabolic profiling, tracking, and tumor single-cell sequencing in order to accurately stratify patients and implement customized treatments (Gandhi and Das, 2019).
